# How relative deprivation affects the sleep quality of Chinese college students: testing an integrated model of social anxiety and trait mindfulness

**DOI:** 10.3389/fpsyg.2023.1111845

**Published:** 2023-05-18

**Authors:** Meng Xiong, Jiao Chen, Yiduo Ye

**Affiliations:** ^1^School of Education and Sports Sciences, Yangtze University, Jingzhou, China; ^2^Department of Psychology, University of Edinburgh, Edinburgh, United Kingdom; ^3^School of Psychology, Fujian Normal University, Fuzhou, China

**Keywords:** relative deprivation, sleep quality, social anxiety, trait mindfulness, college student

## Abstract

Although previous studies have confirmed the association between relative deprivation and individual health, the relationship between and underlying mechanisms of relative deprivation and sleep quality have rarely been explored. Therefore, the present study investigated how relative deprivation affected sleep quality by testing an integrated model and examining the roles of social anxiety and trait mindfulness. We surveyed 568 college students using the Relative Deprivation Scale, Interaction Anxiousness Scale, Mindful Attention Awareness Scale, and Pittsburg Sleep Quality Index. Data were analyzed using SPSS 24.0 and PROCESS macro for SPSS. We found that a high relative deprivation score predicted poor sleep quality, and social anxiety partially mediated this relationship. Our model also indicated that the relationship between relative deprivation and sleep quality via social anxiety was moderated by mindfulness. Specifically, increasing trait mindfulness may decrease the indirect effect of relative deprivation on sleep quality through social anxiety. The current study expands our understanding of the underlying mechanisms, paths, and conditions of the effects of relative deprivation on sleep quality. Furthermore, we provide additional evidence that trait mindfulness can mitigate the adverse effects of negative events. College students should consciously use trait mindfulness techniques to consider problems, reduce anxiety, and improve their sleep quality.

## Introduction

Sleep plays a crucial role in maintaining human health; people spend almost one-third of their lives asleep (Wang et al., 2019). According to the Report on Mengjie Big data research (2020), approximately 300 million people had sleep quality problems, and nearly 67.24% of those surveyed experienced insomnia. Notably, insomnia and other sleep problems are quite common among young adults, especially college students (Samaranayake et al., 2014; Jiang et al., 2015). Some studies have proven that nearly 70% of college students report insufficient sleep (Fu and Zhang, 2021). Poor sleep quality is a crucial public health problem that increases the risk of premature morbidity and mortality (Stranges et al., 2012). Specifically, poor sleep quality can cause a variety of undesirable consequences such as poor academic performance (Maheshwari and Shaukat, 2019), dietary risk (Du et al., 2021), suicidal ideation (Littlewood et al., 2019), and other mental health problems (Kim et al., 2021). Therefore, it is important to identify and characterize the factors that modulate sleep quality and quantity among college students. While sleep problems among college students are becoming increasingly serious, with the rapid growth of China’s economy and due to the widening gap between the rich and the poor and changes in social status, college students are also psychologically prone to a sense of relative deprivation (Xiong and Ye, 2016).

A substantial body of literature has established links between negative emotions and poor sleep quality (Shen et al., 2018; Wołyńczyk-Gmaj et al., 2022). Relative deprivation has a negative emotional component (Smith et al., 2012); hence, it may also be associated with sleep quality in college students. A previous study on relative deprivation and mental health also showed that relative deprivation was positively associated with relevant items for poor sleep quality in adults (Callan et al., 2015). However, the underlying mediating and moderating processes in this relationship among college students remain unclear. Numerous studies have shown that trait mindfulness and social anxiety are associated with individual sleep quality (Bogusch et al., 2016; Burrowes et al., 2022). Specifically, trait mindfulness is negatively correlated with poor sleep quality (Liu et al., 2018), whereas social anxiety is positively correlated with poor sleep quality (Horenstein et al., 2019). Therefore, this study constructed an integrated model to test the mediating role of social anxiety and the moderating role of trait mindfulness in the relationship between relative deprivation and sleep quality. The findings would promote our understanding of how and when relative deprivation is associated with college students’ sleep quality.

## Theory and hypotheses

### Relative deprivation and sleep quality

Relative deprivation refers to a subjective cognition and emotional experience in which an individual or group perceives itself to be in a disadvantageous position through horizontal or vertical comparisons with the reference group, which leads to negative emotions such as anger and dissatisfaction (Crosby, 1976; Smith et al., 2012). According to the 3-P model of insomnia, sleep is influenced by predisposing, precipitating, and perpetuating factors (Spielman et al., 1987). Therefore, the experience of adverse events or bad behavioral tendencies (upward social comparison) will damage the physical and mental health of individuals, resulting in poorer sleep quality (Zhang and Liu, 2022). The core process of relative deprivation is upward social comparison (bad behavior tendency), which is one of the factors influencing insomnia (Appelgryn and Bornman, 1996; Kim et al., 2018). According to the theory of sleep-interfering processes, excessive emotional arousal, such as negative emotions, interferes with the normal sleep process and affects sleep quality (Lundh and Broman, 2000; Rash et al., 2019). Relative deprivation includes negative emotions, such as anger and dissatisfaction, which may worsen sleep quality (Smith et al., 2012). Previous sub-studies on the relationship between relative deprivation and individual health also showed that relative deprivation positively predicted poor sleep quality; that is, the higher the level of relative deprivation, the worse the individual’s sleep quality (Callan et al., 2015). Therefore, we hypothesized that relative deprivation would be positively correlated with poor sleep quality in college students (Hypothesis 1).

### Mediating role of social anxiety

Social anxiety typically involves intense fear of others’ evaluations of our social interactions (American Psychiatric Association, 2013; Morrison and Heimberg, 2013). The core process of relative deprivation is upward social comparison, whereby individuals perceive themselves as disadvantaged and inferior to others, which may also lead to negative self-evaluations (Osborne et al., 2015; Kim et al., 2018; Kuo and Yang, 2019). According to the cognitive self-assessment model of social anxiety, the root of social anxiety lies in negative self-evaluations after social interactions (Heinrichs and Hofmann, 2005; Amiri et al., 2017). In addition, the cognitive dissonance model of social anxiety states that social anxiety is the result of cognitive dissonance (Young, 1986) and that relative deprivation includes the cognitive components of individuals in disadvantageous positions (Smith et al., 2012). Moreover, two studies of vulnerable Chinese children demonstrated that the relative deprivation of migrant and single-parent children could positively predict their social anxiety (Xiong et al., 2021a,b). While the extension of this result to college students is yet to be confirmed, it provides direct support for the positive prediction of social anxiety by relative deprivation.

Furthermore, social anxiety, as an important indicator of mental health, is closely related to sleep quality; participants with social anxiety disorder reported poorer sleep quality than healthy participants did (Horenstein et al., 2019; Mohammadi et al., 2020). The maintenance cognitive model of insomnia highlights that anxiety states trigger the individual’s selective attention and monitoring of internal and external sleep-related threat cues, leading to an overestimation of the extent of the perceived sleep deficit and daytime performance reduction (Harvey, 2002). In other words, when individuals experience social anxiety, sleep-related threats, and sleep deprivation are more easily perceived and excessive negative cognitive activity is activated, which exacerbates insomnia and reduces sleep quality (Hiller et al., 2015). According to the 3-P model of insomnia, anxiety is also a predisposing factor, further making an individual prone to sleep problems (Bélanger et al., 2016). Previous studies have shown a positive correlation between social anxiety and poor sleep quality (Wang et al., 2017; Lima et al., 2020).

Moreover, Harvey’s cognitive model for overcoming insomnia provides a conceptual framework for the association between relative deprivation, social anxiety, and poor sleep quality (Harvey, 2002; Hiller et al., 2015). While excessive negative cognitive activity (e.g., relative deprivation) is central to the model, other components may also directly exacerbate or indirectly influence the factors that contribute to perceived sleep deficits. These components include arousal, distress, selective attention, and monitoring (Woodley and Smith, 2006). Specifically, individuals experience a sense of relative deprivation that they are at a disadvantage of negative cognition, which may arouse negative emotions of social anxiety (Xiong et al., 2021a,b), thereby causing them to focus on and monitor their disadvantaged position; this increases their anxiety. Social anxiety exacerbates the adverse effects of relative deprivation, which, in turn, has a cumulative negative effect on sleep quality. Moreover, prior studies have found that social anxiety plays an intermediary role in family functioning and migrant adolescents’ sleep quality (Wang et al., 2017). Therefore, social anxiety may also act as an intermediary between relative deprivation and poor sleep quality. Given the above, we investigated whether social anxiety plays an intermediary role between relative deprivation and poor sleep quality in college students (Hypothesis 2).

### Moderating role of trait mindfulness

Although relative deprivation may affect college students’ social anxiety and sleep quality, not all college students may be equally vulnerable. Thus, it is necessary to consider moderators that may affect the relationship between relative deprivation and social anxiety and between relative deprivation and sleep quality. Trait mindfulness is a positive individual quality factor that refers to an individual’s habitual awareness of current thinking and emotions, including observing experience, marking perception with words, being aware of the present moment, not judging one’s own thoughts and emotions, and not overreacting (Brown and Ryan, 2003; Baer et al., 2004). Previous studies have shown that trait mindfulness is negatively correlated with individual anxiety; that is, the higher an individual’s trait mindfulness, the lower their social anxiety (Makadi and Koszycki, 2020; Liu et al., 2021). The reperceiving model of mindfulness states that trait mindfulness can help individuals objectively re-perceive their moment-by-moment experiences, eliminate unconscious behavioral and emotional patterns, and promote adaptive responses to negative stimuli (Shapiro et al., 2006). In other words, high trait mindfulness encourages individuals to utilize adaptive withdrawal, objective experience, and inclusive acceptance of adverse factors to alleviate the negative effects of such factors (Chen et al., 2011). Therefore, when individuals experience relative deprivation, high trait mindfulness can cause them to withdraw from their current state and become more objective, thereby minimizing the impact of relative deprivation on social anxiety.

The conservation of resources model proposes that when an individual is under pressure or threat, the strategy of introducing resources is usually employed as a buffer against the threat so that they can reach a state of conservation (Hobfoll, 1989; Hagger, 2015). Previous studies have suggested that mindfulness traits play a significant role in promoting the construction of individual psychological resources, which can be used as a buffer against adverse events (Garland et al., 2015). Therefore, the introduction of trait mindfulness as a psychological resource can effectively alleviate the negative impact of relative deprivation on individuals by reducing social anxiety. In addition, previous studies have shown that trait mindfulness not only plays a positive role in promoting individual psychosocial adaptation but also plays a protective role in the impact of negative factors on individual physical and mental health (e.g., anxiety; Bajaj et al., 2016; Davis et al., 2016). For example, trait mindfulness moderates the relationship between mobile phone addiction and anxiety in adolescents. For high-trait mindfulness individuals, mobile phone addiction has a small positive predictive effect on adolescent anxiety (Yang et al., 2019). Therefore, trait mindfulness, as a positive personality trait, may buffer the detrimental outcomes of relative deprivation (e.g., social anxiety and poor sleep quality). Based on the above, we hypothesized that trait mindfulness would moderate the indirect effect of relative deprivation on sleep quality (Hypothesis 3).

### The current study

**Figure 1:**
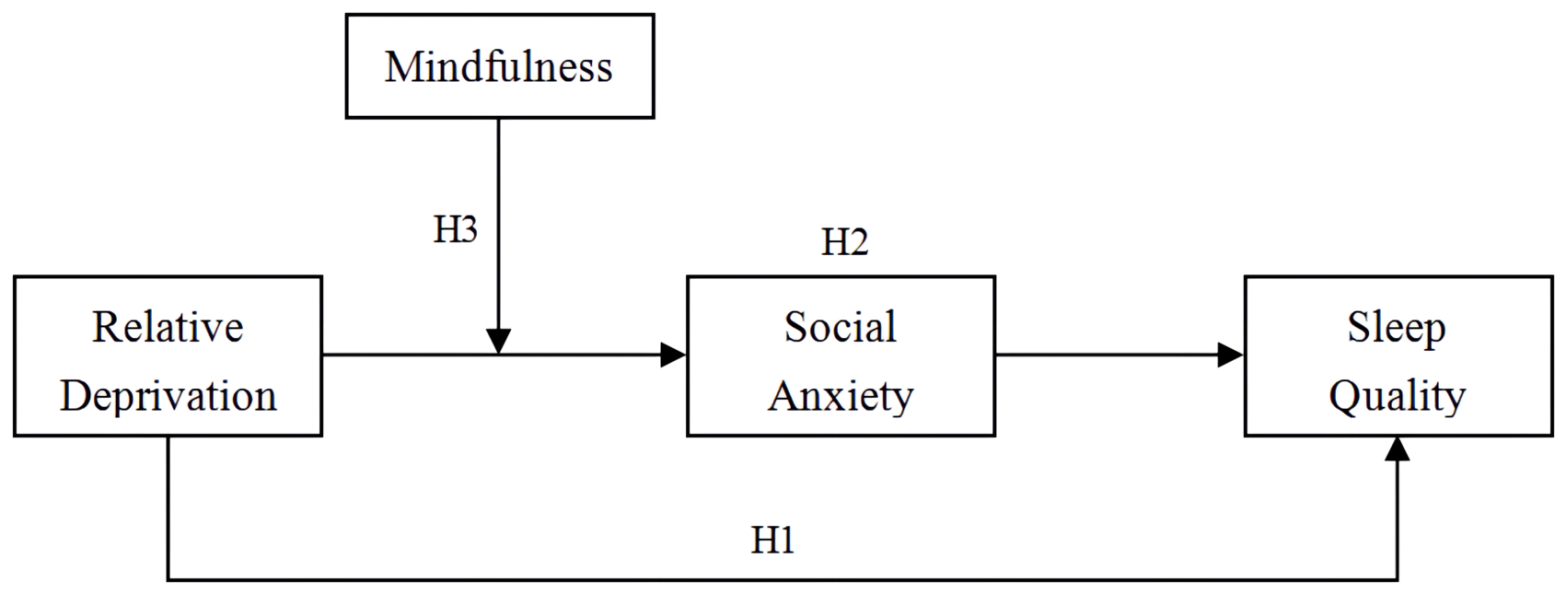
Moderated mediation model of the present study.

This study explored the mechanisms underlying the association between relative deprivation and sleep quality among Chinese college students. Specifically, we examined a moderated mediation model (see Figure 1) to test three hypotheses: relative deprivation positively correlates with poor sleep quality among Chinese college students (Hypothesis 1); social anxiety plays an intermediary role between relative deprivation and poor sleep quality among Chinese college students—that is, relative deprivation is positively associated with social anxiety, which, in turn, is positively associated with poor sleep quality (Hypothesis 2); and trait mindfulness moderates the indirect effect of relative deprivation on poor sleep quality via social anxiety (Hypothesis 3). Moreover, significant sex and age differences have been observed in relative deprivation (Leviston et al., 2020), social anxiety (Scaini et al., 2014; Asher and Aderka, 2018), and sleep quality (Bruni et al., 2015; Meers et al., 2019); thus, sex and age were used as control variables in this study.

Overall, this study makes the following contributions. First, previous studies have rarely explored the relationship between individual relative deprivation and sleep quality. In the few studies that explored the relationship between the two, only three items (sleep latency) in the Pittsburgh Sleep Quality Scale were used to measure sleep quality, with few items and incomplete measurement (Callan et al., 2015). This study measured all 19 items of the scale, including subjective sleep quality, sleep latency, sleep duration, habitual sleep efficiency, sleep disturbances, use of sleep medications, and daytime dysfunction (Buysse et al., 1989). That is, this is a comprehensive study on the relationship between relative deprivation and sleep quality. Second, previous studies may have investigated the relationship among these variables separately; however, we built a moderating mediation model to integrate different variables, which is conducive to exploring the processes by which these variables impact sleep quality. Third, this study provides additional evidence that trait mindfulness can mitigate the adverse effects of negative events. Fourth, this study provides guidance to improve sleep quality.

## Materials and methods

### Participants and procedures

This study was approved by the Ethical Committee of Academic Research at the corresponding author’s institution. We recruited participants from a full-time college in central China using convenience sampling. The final sample comprised 568 college students with a mean age of 19.98 years (*SD* = 1.66, range 16–26). Of these, 254 (44.7%) were men, and 314 (55.3%) were women. The sample size was selected with reference to previous studies and was calculated strictly according to the formula ( $n=\frac{Z^{2}pq}{e^{2}}=\frac{1.96^{2}*0.5*0.5}{0.05^{2}}$ ) (Gegez, 2007; where “*n*” represents the sample size, “*Z*” represents the statistic of confidence level, “*p*” represents the possibility of options, and “*e*” represents sampling error), resulting in a final minimum sample size of 385 (Lwanga and Lemeshow, 1991). The effective sample size of this study was greater than 385, which was acceptable.

Questionnaire surveys in paper-and-pencil format were conducted in different classrooms during the 20-min class period. In each classroom, two trained psychology graduate students administered the surveys, provided clarifications where needed, and monitored participants’ progress. Informed consent was obtained from the participants and their teachers before the questionnaire survey was conducted. To ensure and improve the validity and reliability of this study, we carefully designed its procedures. For instance, the wording of some items was reversed to motivate participants to provide unbiased answers (Mackenzie and Fowler, 2013). After completing the questionnaire, each participant was gifted a small jotter.

### Measures

#### Relative deprivation

Relative deprivation was measured using the Relative Deprivation Scale (RDS; Ma, 2012), which includes four items (e.g., “Comparing my efforts with those of others, my life should have been better” and “I always think that others have something that should belong to me”). Participants responded on a six-point Likert scale ranging from 1 (*strongly disagree*) to 6 (*strongly agree*). Higher scores represented higher levels of RD. This scale has been used in previous studies and has been demonstrated to have good reliability and validity (Liu et al., 2021; Ye et al., 2021). In this study, Cronbach’s α for the scale was 0.72.

#### Social anxiety

We assessed college students’ social anxiety using the Chinese version of the Interaction Anxiousness Scale (IAS; Leary and Kowalski, 1993). The IAS includes 15 items (e.g., “I feel nervous even at informal parties”), of which four items are reverse-scored (e.g., “I am probably less shy in social interactions than most people are”). Participants were instructed to rate the extent to which each item described them. A five-point Likert scoring system was applied to the IAS, where 1 = “*not at all*” and 5 = “*extremely.*” A high score for adolescent students indicates a high level of subjective interaction anxiety. This scale has been used in previous studies and has demonstrated good reliability and validity (Buyukbayraktar, 2020; Zhou et al., 2022). In this study, the Cronbach’s α for the IAS was 0.88.

#### Trait mindfulness

We used the Chinese version of the Mindful Attention Awareness Scale (MAAS; Brown and Ryan, 2003), which includes 15 items (e.g., “I could be experiencing some emotion and not be conscious of it until sometime later,” and “I forget a person’s name almost as soon as I’ve been told it for the first time”). MAAS respondents indicate how frequently they encounter the experience described in each statement using a six-point Likert scale, where 1 = “*almost always*” and 6 = “*almost never*”; higher scores reflect more trait mindfulness. This scale has been used in previous studies and has demonstrated good reliability and validity (Yue et al., 2020). In the current study, the Cronbach’s α for this scale was 0.88.

#### Sleep quality

The Chinese version (Liu et al., 1996) of the Pittsburg Sleep Quality Index (PSQI; Buysse et al., 1989) was used in our study. It includes 18 items (e.g., “During the past month, how many minutes did it usually take you to sleep every night?” and “During the past month, how would you rate your overall sleep quality?”) to measure seven aspects of sleep quality (e.g., sleep latency, sleep efficiency, and sleep duration). Accordingly, these 18 items were grouped into seven component scores, each weighted equally on a 0–3 point scale. The seven component scores were then summed to yield a global PSQI score, with a range of 0–21; higher scores indicate poor sleep quality (thus labeled as poor sleep quality in the Results section). The PSQI has been widely used in Chinese college student samples and is highly reliable and valid (Li et al., 2019). The Cronbach’s α for the Chinese version of the PSQI used in this study was 0.68.

### Statistical analyses

All statistical analyses were conducted using IBM SPSS for Windows version 24.0. We first calculated the descriptive statistics and variable correlations. Next, we used Model 4 in PROCESS macro (Hayes and Scharkow, 2013) to test the mediation effect and Model 7 to test the moderated mediation. This macro was developed to test complex models, including both mediators and moderators, and has been extensively used in previous research (Gori et al., 2021; Borah et al., 2022). In addition, considering that college students’ sex and age are associated with relative deprivation, social anxiety, and sleep quality (Asher and Aderka, 2018; Meers et al., 2019; Leviston et al., 2020), we included sex and age as covariates in the statistical analyses. Moreover, we used Harman’s one-factor test for all research items to test potential common method biases (Podsakoff et al., 2003, 2012). The results revealed the presence of nine distinct factors with eigenvalues greater than 1, with the largest factor accounting for 15.23% of the variance (<40%, the threshold level; Zhou and Long, 2004). Therefore, we conclude that no common method bias was apparent in the present study.

## Results

### Preliminary analyses

Descriptive statistics and the Pearson’s correlation matrix are presented in Table 1. As hypothesized, relative deprivation was negatively correlated with trait mindfulness (*r =* −0.15, *p* < 0.01) and positively correlated with poor sleep quality (*r =* 0.16, *p* < 0.01) and social anxiety (*r =* 0.24, *p* < 0.01). Social anxiety was positively correlated with poor sleep quality (*r =* 0.15, *p* < 0.01). The key variables were moderately correlated.

**Table 1:** Descriptive statistics and correlations among key variables.

Variables	*Mean*	*SD*	1	2	3	4	5	6
1. Age	19.98	1.66	−					
2. Sex	/	/	−0.04	−				
3. Relative deprivation	3.05	0.95	−0.05	−0.13^**^	−			
4. Mindfulness	3.32	0.72	−0.08^*^	0.05	−0.15^**^	−		
5. Social anxiety	3.20	0.58	−0.03	0.02	0.24^**^	0.03	−	
6. Sleep quality	5.30	2.63	0.01	−0.04	0.16^**^	0.05	0.15^**^	−

*N* = 568. ^*^p < 0.05; ^**^p < 0.01.

Independent-sample *t*-tests (Table 2) showed that relative deprivation (*t =* 3.13, *p* < 0.01, Cohen’s *d* = 0.26) and trait mindfulness (*t =* −1.17, *p* < 0.05, Cohen’s *d* = 0.097) were significantly associated with sex differences. Specifically, the social anxiety of women was significantly lower than that of men. By contrast, the trait mindfulness of women was significantly higher than that of men.

**Table 2:** Independent sample *t*-test of sex on key variables.

Variables	Male		Female		*t*	*p*	*Cohen’s d*
	*Mean*	*SD*	*Mean*	*SD*			
Relative deprivation	3.19	1.04	2.94	0.86	3.13^**^	**0.007**	**0.26**
Mindfulness	3.28	0.79	3.35	0.65	−1.17^*^	**0.019**	**0.097**
Social anxiety	3.19	0.60	3.21	0.56	−0.38	0.459	0.034
Sleep quality	5.40	2.73	5.21	2.56	−0.83	0.468	0.072

*N* = 568. ^*^p < 0.05; ^**^p < 0.01.

### Testing the mediation model

We used a bootstrap sample of 5,000, below the 95% confidence interval. After controlling for age and sex (Table 3), relative deprivation was positively associated with poor sleep quality (*B* = 0.15, *t* = 3.55, *p* < 0.001, 95% CI [0.07, 0.23]) and social anxiety (*B* = 0.23, *t* = 2.61, *p* < 0.001, 95% CI [0.15, 0.31]). Social anxiety was positively associated with poor sleep quality (*B* = 0.11, *t* = 2.61, *p* < 0.01, 95% CI [0.03, 0.20]). Furthermore, relative deprivation remained positively associated with poor sleep quality (*B* = 0.12, *t* = 2.87, *p* < 0.01, 95% CI [0.04, 0.21]) when both relative deprivation and social anxiety were included in the model, suggesting that social anxiety partially mediated the relationship between relative deprivation and poor sleep quality (indirect effect = 0.03, *SE* = 0.01, 95% CI [0.01, 0.05]). Specifically, the mediating effect accounted for 20% of the total variance. Thus, Hypotheses 1 and 2 are supported.

**Table 3:** Testing mediation of social anxiety between relative deprivation and sleep quality.

Outcome (Y)	Predictors (X)	Model summary					95% CI
		*R*	*R*^2^	*F*	*B*	*SE*	
SQ		0.19	0.04	5.11^***^			
	Age				0.01	0.03	[−0.04,0.06]
	Sex				−0.03	0.08	[−0.20, 0.13]
	SC				−0.12^*^	0.06	[−0.23, −0.01]
	RD				0.15^***^	0.04	[0.07, 0.23]
SA		0.26	0.07	10.43^***^			
	Age				−0.02	0.02	[−0.07, 0.031]
	Sex				0.09	0.08	[−0.07, 0.25]
	SC				−0.12^*^	0.05	[−0.23, −0.02]
	RD				0.23^***^	0.04	[0.15, 0.31]
SQ		0.22	0.05	5.5^***^			
	Age				0.01	0.03	[−0.04, 0.06]
	Sex				−0.04	0.08	[−0.21, 0.12]
	SC				−0.10	0.06	[−0.21, 0.005]
	RD				0.12^**^	0.04	[0.04, 0.21]
	SA				0.11^**^	0.04	[0.03, 0.20]

Effect	*B*	Boot SE	Boot LLCI	Boot ULCI
Direct	0.12	0.04	0.04	0.21
Indirect	0.03	0.01	0.01	0.05

*N* = 568. Bootstrap sample size = 5,000. CI, confidence interval; LL, low limit; UL, upper limit; SC, subjective class; RD, relative deprivation; SA, social anxiety; SQ, sleep quality.

^*^p < 0.05; ^**^p < 0.01; ^***^p < 0.001.

### Testing the moderated mediation model

**Figure 2:**
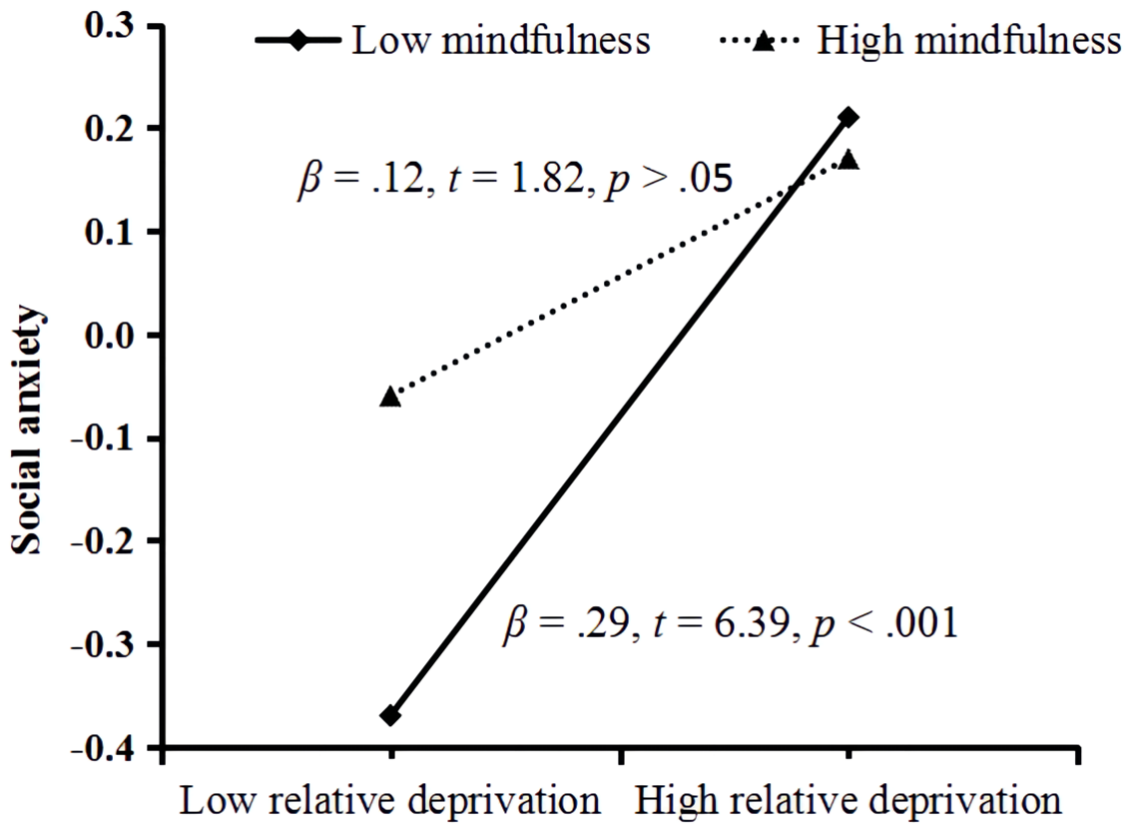
Plot of the relationship between relative deprivation and social anxiety at two levels of trait mindfulness.

Table 4 presents the results of the moderated mediation analysis. We used a bootstrap sample of 5,000, below the 95% confidence interval. We observed that the relationship between relative deprivation and social anxiety was moderated by trait mindfulness (*B* = −0.09, *t* = 0.03, *p* < 0.01, 95% CI [−0.15, −0.02]). To test and illustrate the moderating effect more clearly, we conducted simple slope analyses (Aiken and West, 1991; pick-a-point approach) separately for low (1 SD below the mean = 2.6; trait mindfulness*_M_* = 3.32) and high (1 SD above the mean = 4.04) trait mindfulness. As shown in Figure 2, for college students with low trait mindfulness, relative deprivation significantly positively predicted social anxiety (*β*_simple slope_ = 0.29, *t =* 6.39, *p* < 0.001), whereas for college students with high trait mindfulness, relative deprivation was not a significant predictor of social anxiety (*β*_simple slope_ = 0.12, *t =* 1.82, *p* > 0.05).

**Table 4:** Moderated mediation model with mindfulness as moderator.

Outcome (Y)	Predictors (*X*)	Model summary			*B*	*SE*	95% CI
		*R*	*R*^2^	*F*			
SA		0.29	0.08	8.58^***^			
	Age				−0.01	0.02	[−0.06, 0.04]
	Sex				0.09	0.08	[−0.07, 0.25]
	SC				−0.12	0.05	[−0.22,-0.01]
	RD				0.20^***^	0.04	[0.11, 0.29]
	M				0.07	0.04	[−0.01, 0.15]
	RD × M				−0.09^**^	0.03	[−0.15, −0.02]

Effect	*M* values	*B*	Boot SE	Boot LLCI	Boot ULCI
	*M*-1*SD* (−1.004)	0.29	0.05	0.20	0.38
Indirect	*M* (−0.003)	0.20	0.04	0.12	0.29
	*M* + 1*SD* (0.999)	−0.12	0.06	−0.01	0.24

*N* = 568. Bootstrap sample size = 5,000. CI, confidence interval; LL, low limit; UL, upper limit; SC, subjective class; RD, relative deprivation; M, mindfulness; SA, social anxiety.

^*^p < 0.05; ^**^p < 0.01; ^***^p < 0.001.

**Figure 3:**
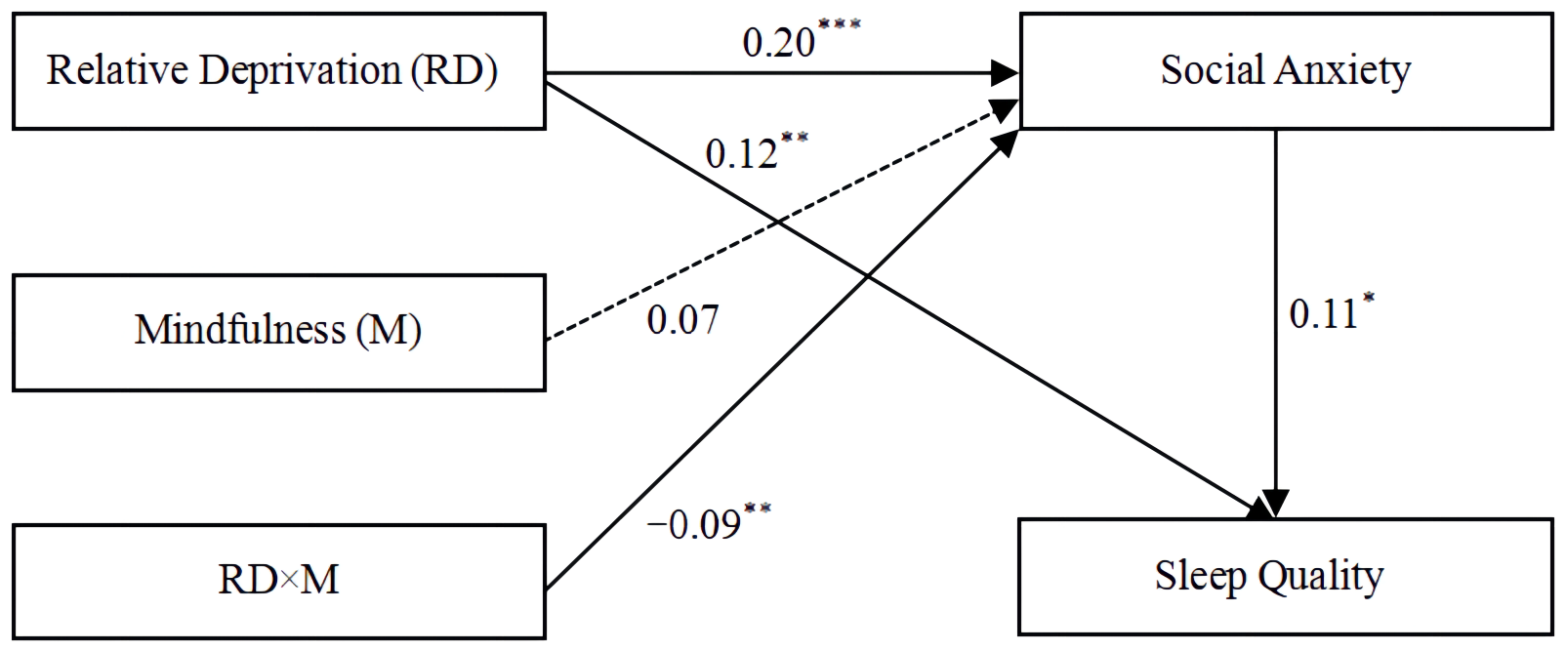
Moderated mediation model with key results for college students.

Moreover, the conditional indirect effect test indicated that trait mindfulness moderated the indirect effect of relative deprivation on sleep quality through social anxiety. Specifically (see Table 4), for college students with lower trait mindfulness scores, relative deprivation significantly affected sleep quality via social anxiety (*B* = 0.03, SE = 0.01, *p* < 0.01, 95% CI [0.01, 0.06]). However, for college students with stronger trait mindfulness (*M* > 4.04), this indirect effect was not significant (*B* = 0.01, SE = 0.01, *p* > 0.05, 95% CI [−0.002, 0.04]). Therefore, Hypothesis 3 is supported. Figure 3 intuitively describes the moderated mediation model and its key path coefficients for college students.

**Figure 4:**
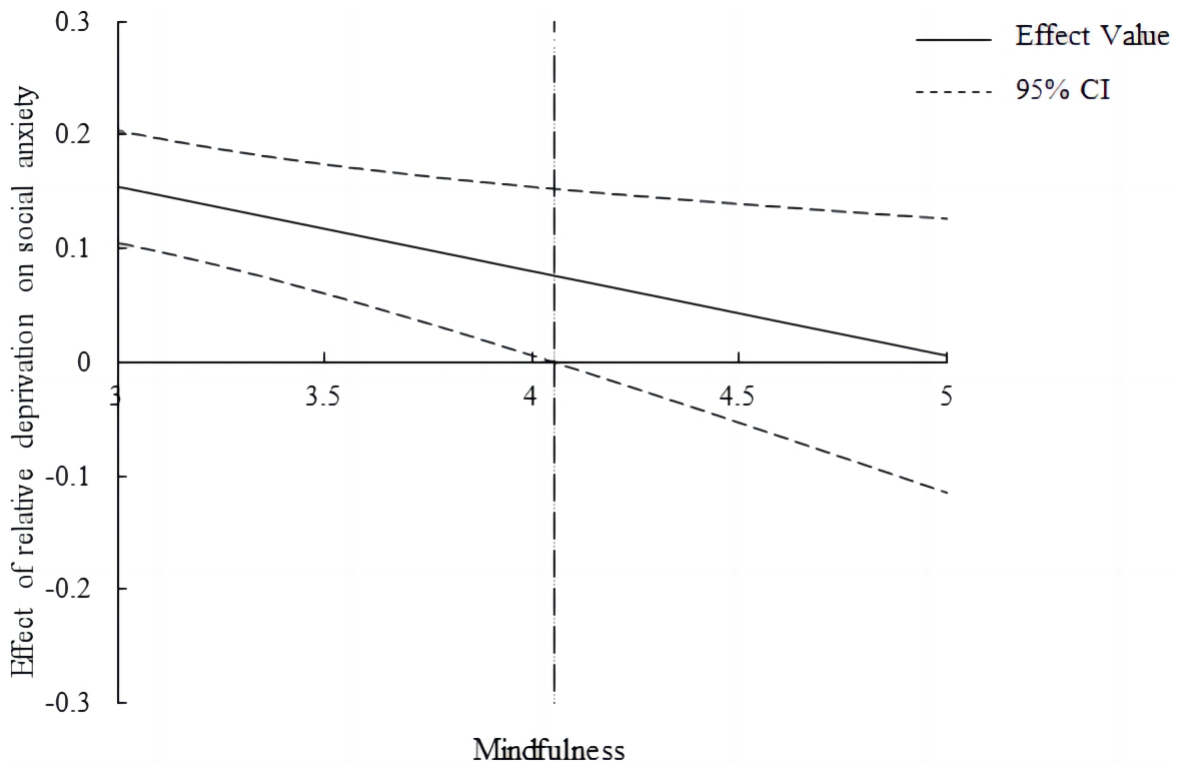
Effect of relative deprivation on social anxiety with Johnson–Neyman confidence bands.

Furthermore, we used the Johnson–Neyman technique to identify regions in the range of the moderator variable where the indirect effect of relative deprivation on sleep quality through social anxiety was statistically significant and those where it was not significant (Hayes, 2017; Sun et al., 2021), as shown in Figure 4. The effect of relative deprivation on social anxiety was significant within a range of the scores for trait mindfulness (4.05). For college students with lower trait mindfulness, the effect of relative deprivation on social anxiety was stronger. However, for college students with stronger trait mindfulness (*M* > 4.05), this indirect effect was not significant. This result is basically consistent with the pick-a-point approach, which further verifies our moderating effect analysis.

## Discussion

We constructed a moderated mediation model to test three hypotheses regarding the psychological processes underlying the relationship between relative deprivation and sleep quality among college students. Social anxiety mediated and mindfulness moderated this relationship. Specifically, the indirect effect of relative deprivation on poor sleep quality via social anxiety was greater among college students with lower trait mindfulness scores than among those with higher scores. These findings promote our understanding of how and when relative deprivation is associated with poor sleep quality among college students.

### Relative deprivation and sleep quality

The present study found a significant positive correlation between relative deprivation and poor sleep quality, implying that the higher the level of relative deprivation, the worse the sleep quality of college students. This finding is consistent with previous research (Callan et al., 2015), demonstrating that relative deprivation is positively correlated with poor sleep quality. It is also congruent with the 3-P model of insomnia (Zhang and Liu, 2022), which states that the core component of relative deprivation, as one of the factors affecting insomnia, is upward social comparison, which lowers sleep quality and thus adversely affects health. Moreover, this conclusion further verifies the theory of sleep-interfering processes (Ong et al., 2012). Negative emotions such as anger and resentment in relative deprivation instigate in individuals a state of excessive emotional awakening, which interferes with their normal sleep and leads to problems such as insomnia (Lundh and Broman, 2000; Smith et al., 2012). Our study found a consistent relationship between relative deprivation and sleep quality, and the research sample was focused on college students. The measurement index of sleep quality was also more comprehensive, which has been further verified and expanded in previous studies.

### Mediating effect of social anxiety

Our models indicated that relative deprivation was both directly and indirectly associated with sleep quality through the mediating role of social anxiety. Previous studies have indicated that social anxiety is an important mediating variable in the relationship between upward social comparison (the core component of relative deprivation) and mental health (Zhang and Liu, 2022). For instance, recent research has indicated that the relationship between relative deprivation and interpersonal adaptability in college freshmen is partially mediated by social anxiety (Zhang et al., 2022). Regarding the first part of the mediation effect, higher levels of relative deprivation were associated with higher levels of social anxiety among college students, which is consistent with the findings of previous studies (Xiong et al., 2021a,b). This result is consistent with the cognitive evaluation model of social anxiety (Glass et al., 1982). When individuals experience relative deprivation through upward comparison and perceive themselves to beat a disadvantage, they will lower their self-evaluation and consider themselves to be worse than others, thus forming a negative cognitive evaluation of themselves (Kuo and Yang, 2019). The core source of social anxiety is the negative evaluation of oneself; therefore, the negative evaluation caused by relative deprivation is influenced by social anxiety (Iverach et al., 2017). Simultaneously, it further verifies the cognitive dissonance theory of social anxiety; owing to the unreasonable cognition contained in the sense of relative deprivation, individuals are prone to social anxiety due to cognitive dissonance (Covey, 2009).

Regarding the second part of the mediation effect, higher levels of social anxiety were associated with poorer sleep quality in college students, which is consistent with prior studies (Mohammadi et al., 2020). This result further validates the cognitive maintenance model of insomnia (Harvey, 2002), wherein an individual’s anxiety triggers their attention and monitoring of their negative emotions. This amplifies the adverse consequences of anxiety and excessive worry about the adverse effects of insomnia, leading to further sleep deprivation, insomnia, and other sleep problems (Hiller et al., 2015). Simultaneously, the cognitive maintenance theory of insomnia provides a conceptual framework for the relationship between relative deprivation, social anxiety, and poor sleep quality. Individuals who experience relative deprivation indulge in negative evaluations and cognitions of themselves, which awaken negative emotions such as social anxiety (Nadler et al., 2020), leading to the accumulation of multiple negative emotions and ultimately producing a greater negative impact on the individual’s sleep quality.

### Moderating effect of trait mindfulness

Our models also indicated that the relationship between relative deprivation and sleep quality via social anxiety was moderated by trait mindfulness. Specifically, the relationship was stronger among college students with low trait mindfulness; thus, trait mindfulness appears to alleviate the indirect impact of relative deprivation on sleep quality through the mediation of social anxiety. These results are consistent with previous studies that observed similar protective roles of trait mindfulness across other facets of mental health, such as anxiety and depression (Watkins and Ohannessian, 2022). For example, studies have shown that trait mindfulness moderates the relationship between mobile phone addiction, adolescent anxiety, and depression. When the level of trait mindfulness is high, the association between mobile phone addiction and adolescent anxiety and depression is weaker; trait mindfulness alleviates the adverse effects of mobile phone addiction on adolescent anxiety and depression (Yang et al., 2019).

The reperceiving model of mindfulness posits that through the process of mindfulness, people will reperceive present-moment experience (e.g., thoughts and feelings) with greater clarity and objectivity (Shapiro et al., 2006). Accordingly, trait mindfulness may promote the adaptive dissociation of one’s current experience, which, in turn, relieves mental health problems, including social anxiety (Norton et al., 2015). The current study also further verified the reperceiving model of mindfulness: trait mindfulness separates individuals from the event itself and helps them automatically perceive thoughts and emotions generated, thereby helping them experience emotions in an objective and rational manner (Shapiro et al., 2006). Thus, individuals develop a clearer and more objective perspective to perceive the occurrence of events. Specifically, when experiencing the negative emotion of relative deprivation, individuals with high trait mindfulness can reduce the negative control of relative deprivation and separate themselves from their negative emotions so that they do not produce automatic habitual responses, including social anxiety, which may affect their sleep quality. However, individuals with low trait mindfulness capabilities are unable to form a stable state of mindfulness to cope with negative emotions and are more likely to be immersed in negative experiences caused by relative deprivation, resulting in automatic responses such as social anxiety and thus poorer sleep quality.

Additionally, our results are consistent with the risk-protective model of resiliency, in which the strength of the relationship between risk factors (e.g., high relative deprivation) and outcomes (e.g., social anxiety) depends on the presence of protective factors (e.g., trait mindfulness), which weaken the adverse effects of risks on outcomes (Zimmerman et al., 1999; Hollister-Wagner et al., 2001). At the same time, this result validates resource conservation theory (Hobfoll, 1989; Park et al., 2014), which states that when individuals are in stressful or threatening situations (e.g., relative deprivation), strategies that introduce resources (e.g., trait mindfulness, a positive psychological resource) are often used to alleviate stress and threats to reduce individual health problems, including social anxiety (Chen et al., 2015).

### Limitations and implications

This study has several limitations. First, it is cross-sectional in nature, which makes it difficult to draw causal inferences. Future studies should use longitudinal methods or experiments to further clarify the causal relationship between relative deprivation and sleep quality. Second, the self-reporting method may affect the accuracy of the results because of biases such as social desirability. Future research can collect data from multiple informants (e.g., students, parents, and teachers). Third, the representativeness of the sample may limit the generalizability of our results, as our participants were from the same college. Future research should be conducted across diverse populations.

Despite these limitations, this study has several theoretical implications. First, this study comprehensively explored the relationship between relative deprivation and sleep quality in college students, expanded the previous research population to college students, and measured sleep quality more comprehensively, thereby enriching research on the relationship between relative deprivation and sleep. Second, this study deepens previous research by examining the psychological mechanisms underlying the link between relative deprivation and sleep quality. This contributes to a better understanding of how and when relative deprivation is related to college students’ sleep quality. Third, the results of this study further verify the relative deprivation theory (Smith et al., 2012), the cognitive evaluation model of social anxiety (Glass et al., 1982), the interference process theory of insomnia (Rash et al., 2019), and the cognitive maintenance model (Harvey, 2002). Fourth, this study provides theoretical guidance on how to reduce social anxiety and improve sleep quality among college students.

This study’s findings have several practical implications. First, the government should enhance publicity and guidance regarding college students’ social status so they can realize they are a well-educated group, which will help reduce their relative deprivation and the adverse effects on sleep quality. Second, considering that social anxiety links relative deprivation and sleep quality, parents should extend their support, encourage them to carry out positive upward comparison, participate in daily interpersonal activities, and improve students’ self-evaluation to reduce their social anxiety and promote sleep quality. Third, trait mindfulness can reduce the impact of relative deprivation on sleep quality by reducing social anxiety in college students. Therefore, schools should carry out mindfulness interventions for students with severe social anxiety or poor sleep quality and regularly conduct state mindfulness training to improve their trait mindfulness and sleep quality. Fourth, college students should also actively participate in mindfulness training, integrate mindfulness thinking into daily life, and make themselves an individual of high trait mindfulness so as to relieve various pressures and improve sleep quality.

## Conclusion

This study constructed a moderated mediation model to explore the relationship between relative deprivation and sleep quality in college students. The results showed that college students’ relative deprivation positively predicted their poor sleep quality. Social anxiety mediated and trait mindfulness moderated the relationship between relative deprivation and social anxiety. Specifically, relative deprivation can lower college students’ sleep quality through social anxiety, but this adverse effect can be buffered by trait mindfulness. When college students’ trait mindfulness level is high, relative deprivation has a lesser predictive effect on social anxiety; that is, relative deprivation has a lesser negative effect on sleep quality through social anxiety. It is necessary, therefore, to carry out trait mindfulness training for college students to reduce the negative impact of adverse factors such as relative deprivation on sleep quality. This study also provides new ideas for future research. Experimental research can be carried out to conduct trait mindfulness interventions for college students to clarify the specific mechanisms of sleep quality.

## Data availability statement

The raw data supporting the conclusions of this article will be made available by the authors, without undue reservation.

## Ethics statement

The studies involving human participants were reviewed and approved by the Ethics Committee of the Academic Research at Yangtze University. Written informed consent to participate in this study was provided by the participants.

## Author contributions

MX planned and implemented the study and collected the data and critically reviewed the draft along with extensive editing. JC interpreted the analyzed data and drafted the manuscript under the supervision of MX and YY. All authors approved the final version of the manuscript for submission.

## Funding

This study was funded by the Major Project for Philosophy and Social Science Research of Hubei Province (no. 19ZD020) and the Key Project of Educational Science Planning of Hubei Province (no. 2022GA028) in China.

## Conflict of interest

The authors declare that the research was conducted in the absence of any commercial or financial relationships that could be construed as a potential conflict of interest.

## Publisher’s note

All claims expressed in this article are solely those of the authors and do not necessarily represent those of their affiliated organizations, or those of the publisher, the editors and the reviewers. Any product that may be evaluated in this article, or claim that may be made by its manufacturer, is not guaranteed or endorsed by the publisher.
